# Discovery of a Magnetic Dirac System with a Large Intrinsic Nonlinear Hall Effect

**DOI:** 10.1021/acs.nanolett.2c04194

**Published:** 2023-01-23

**Authors:** Federico Mazzola, Barun Ghosh, Jun Fujii, Gokul Acharya, Debashis Mondal, Giorgio Rossi, Arun Bansil, Daniel Farias, Jin Hu, Amit Agarwal, Antonio Politano, Ivana Vobornik

**Affiliations:** ‡ CNR-IOM TASC Laboratory, Area Science Park, 34149Trieste, Italy; § Department of Molecular Sciences and Nanosystems, Ca’ Foscari University of Venice, 30172Venice, Italy; ∥ Department of Physics, Northeastern University, Boston, Massachusetts02115, United States; ⊥ Department of Physics, University of Arkansas, Fayetteville, Arkansas72701, United States; # University of Milano, 20133Milano, Italy; ∇ Departamento de Física de la Materia Condensada, Universidad Autónoma de Madrid, 28049Madrid, Spain; ○ Instituto “Nicolás Cabrera” and Condensed Matter Physics Center (IFIMAC), Universidad Autónoma de Madrid, 28049Madrid, Spain; ◆ Department of Physics, Indian Institute of Technology Kanpur, Kanpur208016, India; ¶ Department of Physical and Chemical Sciences, University of L’Aquila, Via Vetoio, 67100L’Aquila, Italy

**Keywords:** nonlinear Hall effect, Dirac antiferromagnet, topology, spin−orbit coupling, ARPES

## Abstract

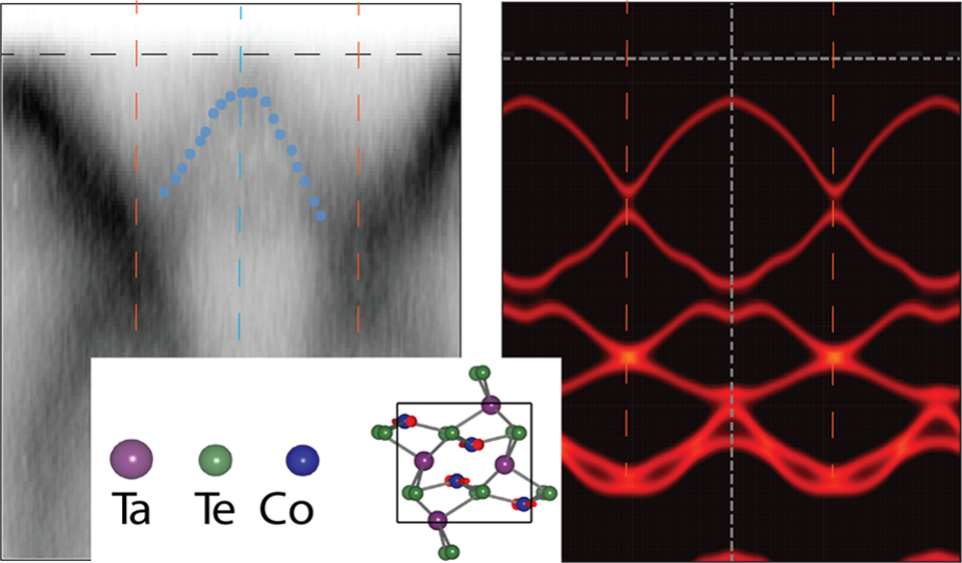

Magnetic materials exhibiting topological Dirac fermions are attracting significant attention for their promising technological potential in spintronics. In these systems, the combined effect of the spin–orbit coupling and magnetic order enables the realization of novel topological phases with exotic transport properties, including the anomalous Hall effect and magneto-chiral phenomena. Herein, we report experimental signature of topological Dirac antiferromagnetism in TaCoTe_2_ via angle-resolved photoelectron spectroscopy and first-principles density functional theory calculations. In particular, we find the existence of spin–orbit coupling-induced gaps at the Fermi level, consistent with the manifestation of a large intrinsic nonlinear Hall conductivity. Remarkably, we find that the latter is extremely sensitive to the orientation of the Néel vector, suggesting TaCoTe_2_ as a suitable candidate for the realization of non-volatile spintronic devices with an unprecedented level of intrinsic tunability.

Over the past few years, magnetic systems with Dirac-like electronic dispersion^1−4^ have been under the spotlight of both theoretical and experimental investigations, because they are expected to support several exotic transport phenomena, including the anomalous and nonlinear Hall effects.^5−7^ In particular, the combined action of magnetic order and spin–orbit coupling (SOC) results in the opening of energy gaps in the electronic spectrum, within which non-trivial topological phases can occur.^8−12^ Such phases, in the presence of long-range magnetic order, can be modulated with ease, thus offering a methodology to tune the topological protection, hence the charges and spins.^4^ Here, we report the experimental discovery of the new system TaCoTe_2_, a topological antiferromagnet with Dirac-like dispersion and SOC-induced gaps at the Fermi level.^13−15^ We discover that the combined effect of SOC and magnetic order enables the realization of a large intrinsic nonlinear Hall effect (INHE), which can be tuned by magnetic fields. Our discovery suggests TaCoTe_2_ as a promising candidate for the realization of highly controllable topological devices that will exploit SOC-derived transport phenomena.^16−19^

Bulk TaCoTe_2_ has a van-der-Waals-type structure with individual monolayers stacked along the [001] crystallographic direction (Figure 1a) with non-symmorphic symmetry^20−23^ (see the Supporting Information for details of growth and characterization). The crystal structure belongs to the monoclinic space group *P*2_1_/*c* (number 14) and gives rise to the constant energy surfaces in reciprocal space shown in panels c and d of Figure 1. Along with the constant energy angle-resolved photoelectron spectroscopy (ARPES) maps collected at 20 meV below the Fermi energy (*E*_F_), we show the Brillouin zone (BZ, blue lines) and the high-symmetry points in Figure 1c. The ARPES spectra were collected with linearly polarized light, i.e., with horizontal (LH) or vertical (LV) polarization (Figure 1b). In the photoelectron excitation process, the photoemission intensity strongly depends upon the light polarization vector direction, in particular, its parity with respect to the mirror plane of the system. In the assumption of an even symmetry for the final state^24−27^, an even (or odd) light polarization vector couples with orbitals with even (odd) parity, to give overall matrix elements even under reflection with respect to the mirror plane. In our case, in a simplistic picture, this means that the light polarization vector couples with those orbitals in the system displaying a non-zero component parallel to the light vector. In particular, LV is only sensitive to the in-plane orbitals, while LH is equally sensitive to in-plane as well as out-of-plane orbitals (given the 45° incidence angle). Using the combination of LH and LV, we conclude that the near *E*_F_ electronic structure is given by an admixture of both in-plane and out-of-plane orbitals, with very little variation in the spectral intensity. This allows us to better identify the spectral features across the BZ and to make a more reliable comparison to the density functional theory (DFT) calculations.

**Figure 1:**
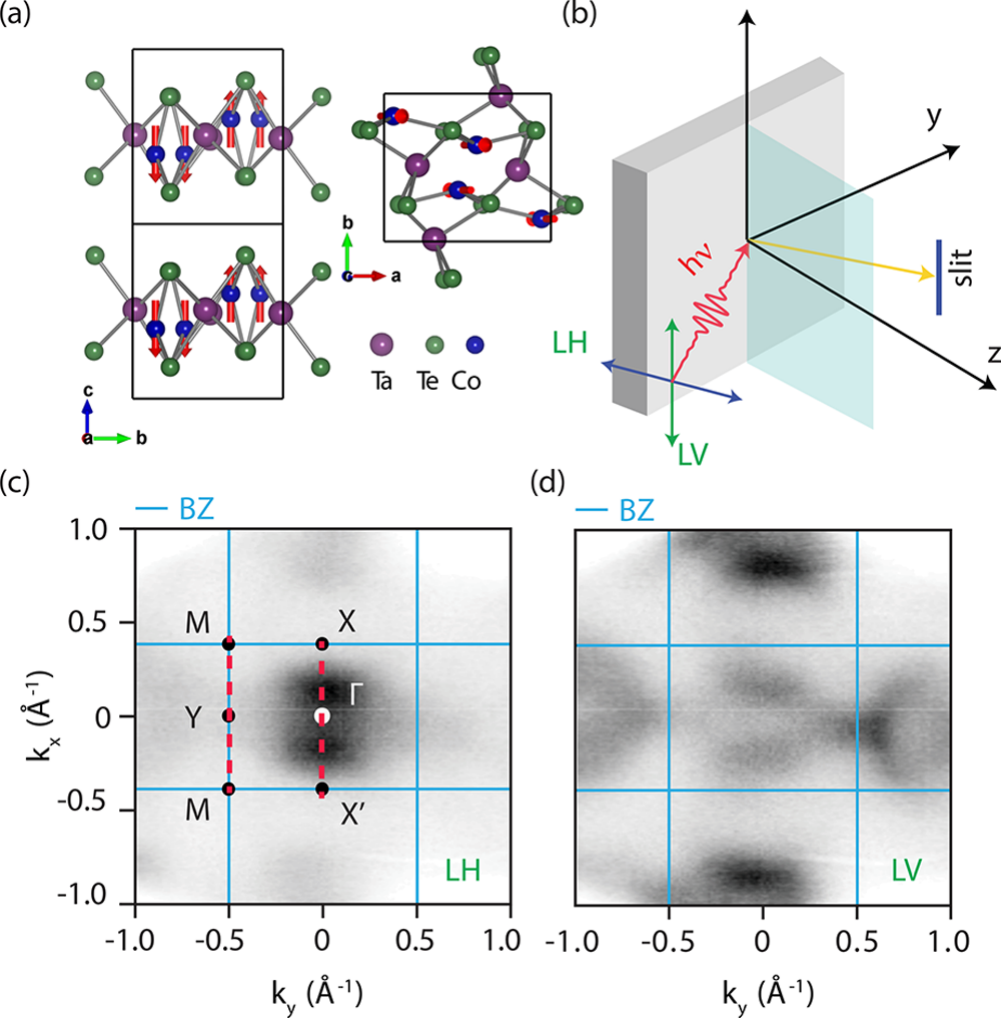
(a) Top and side views of the TaCoTe_2_ crystal structure in the AFM_*z*_ configuration. Red arrows indicate the magnetic moments on the Co atoms. The black solid box identifies the unit cell. (b) Geometry of the experimental setup showing the horizontal (LH) and vertical (LV) light polarization vectors. Fermi surface map collected within 20 meV of the Fermi energy with (c) LH and (d) LV polarization in ARPES at 77 K, with the BZ marked with blue lines. Fermi surface maps reflect the monoclinic crystal structure of TaCoTe_2_, with a different elongation along the *k*_*x*_ and *k*_*y*_ axes.

To understand the experimental electronic structure, we collected energy versus momentum spectra using ARPES. We first notice that the layers of bulk TaCoTe_2_ are weakly bound together by van der Waals forces. Thus, we expect that the electronic structure is intrinsically two-dimensional, with small, if any, electronic dispersion along the *z* direction (perpendicular to the layers). The absence of *k*_*z*_ dispersion in TaCoTe_2_ is confirmed by the qualitatively similar shapes of the spectra at various photon energies^25,28^ (Figure S1 of the Supporting Information). This behavior is fully consistent with a two-dimensional electronic structure. The only change observed experimentally as a function of photon energy is a variation of the spectral intensity across the BZ, likely connected to the photoelectron matrix elements,^26,29^ as also suggested by the data collected for both LH and LV polarization (Figure S2 of the Supporting Information). Our results demonstrate that TaCoTe_2_ behaves electronically as a two-dimensional system; thus, without loss of generality, we will describe the electronic properties of this compound with those of a monolayer.

From DFT, the lowest energy configuration of the system is obtained for magnetic moments on the Co sites arranged in a bicollinear-type antiferromagnetic (AFM) order in each layer (Figure 1a). Such moments are along the *z* direction (AFM_*z*_). The energy difference between this and the AFM_*y*_ order is very small, i.e., 9 meV/unit cell,^15^ but their effect on the electronic structure is sizable, with the realization of markedly different features identifiable in the spectra (as summarized in Figure 2). Both AFM_*z*_ and AFM_*y*_ break the time reversal as well as the inversion symmetry of the system ( and ) while preserving the combined  symmetry, important for the transport and optical properties of the system.^30−32^ The main differences between AFM_*z*_ and AFM_*y*_ are visible in the non-symmorphic symmetry of the system (see the Supporting Information), which manifests directly in different energy versus momentum electronic structures.

**Figure 2:**
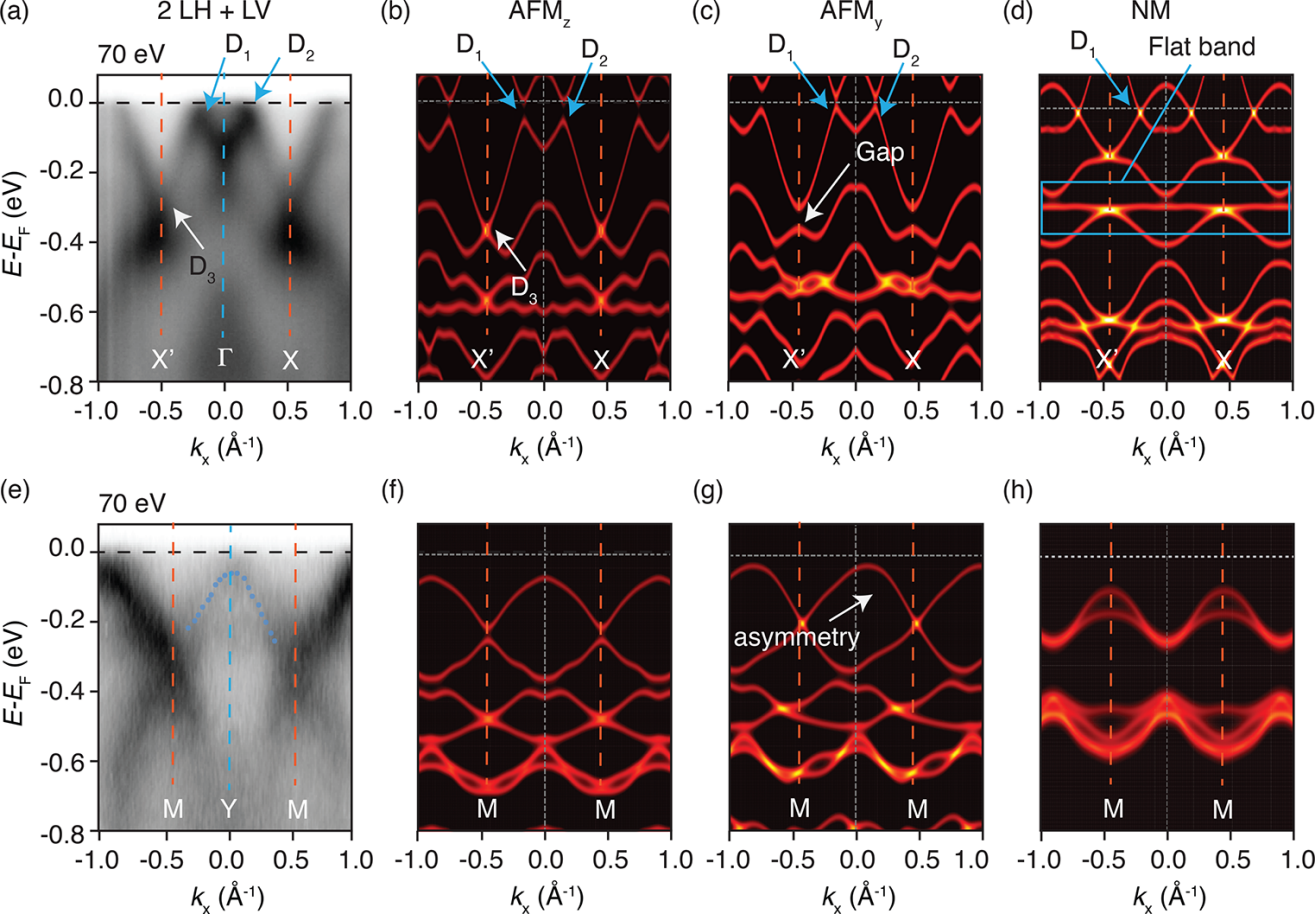
(a) ARPES scan along the X′−Γ–X direction for 70 eV photon energy. Calculated spectral function for monolayer TaCoTe_2_ in the (b) AFM_*z*_, (c) AFM_*y*_, and (d) NM configurations. (e) ARPES map along the M–Y–M direction collected with LV polarization to better see the BZ boundaries and the corresponding DFT calculations for monolayer TaCoTe_2_ in the (f) AFM_*z*_, (g) AFM_*y*_, and (h) NM configurations. Clearly, the theoretical results for the AFM_*z*_ configuration are the closest to the measured ARPES spectrum. Note that the ARPES spectra have been shown as 2LH + LV to account for both light polarizations and also the projections of LH, which is 50% in-plane and 50% out-of-plane. This ensures a better visibility for the spectral features, which might be dependent upon the light polarization.

To unveil the magnetic ordering of this compound, we compared non-magnetic (NM) DFT calculations along with those for the antiferromagnetic AFM_*z*_ and AFM_*y*_ orders, with the measured energy–momentum spectra (Figure 2). Panels a–d of Figure 2 display the results along the high-symmetry direction X′−Γ–X, and panels e–h of Figure 2 display the results for the M–Y–M line, as shown in the BZ of Figure 1c. Evidently, all configurations share common features. Along the X′−Γ–X direction, AFM_*z*_ (Figure 2b), AFM_*y*_ (Figure 2c), and NM (Figure 2d) display Dirac cones located around the Fermi level (labeled D_1,2_). This is also similar to the experiment of Figure 2a. However, a closer inspection reveals important differences: the NM order features a flat band centered around −0.3 eV and a cosine-like dispersion pinned to such a flat band (see the blue guide to the eye). These features are absent in the experiment as well as in the two magnetic calculations. In addition, the NM configuration along the M–Y–M direction (Figure 2h) does not exhibit any band with a maximum at Γ near the Fermi level. On the contrary, this feature is evident in the experimental data of Figure 2e. On the basis of the comparison between ARPES and DFT calculations, one can rule out the NM configuration as the experimental ground state of TaCoTe_2_.

The differences between AFM_*z*_ and AFM_*y*_ along X′−Γ–X are also measured and calculated. Along the M–Y–M path, as a consequence of the magnetism along *y*, the AFM_*y*_ order shifts (about 25%) the maximum of the band at Y toward the M point, creating an extremely large asymmetry in the electronic dispersion (see Figure 2g). This asymmetry, in contrast, is neither observed in the experiment nor predicted in the AFM_*z*_ calculation (Figure 2f). Furthermore, the opening of a gap of a Dirac-like dispersion at −0.3 eV at the X and X′ points for the AFM_*y*_ configuration (indicated as D_3_) is absent in the AFM_*z*_ order and not detected by ARPES, despite its size being expected to be much larger than the experimental resolutions (12 meV and 0.018 Å^–1^ for energy and momentum, respectively). This allows us to conclude that the electronic structure of TaCoTe_2_ is consistent with a magnetic order hosting magnetic moments aligned along the *z* direction, i.e., AFM_*z*_. This is also consistent with our magnetization measurements, which suggest the existence of an easy axis mainly along the out-of-plane direction (Figure S3 of the Supporting Information). Such a magnetization also shows non-trivial magnetic signatures in the curves, i.e., saturating magnetization, which are in full agreement with what was predicted in ref (15). In addition, as mentioned above, AFM_*z*_ is the most stable configuration found for this system. We also notice that ARPES does not resolve the fine details that DFT calculations reveal for binding energies lower than −0.4 eV, regardless of the magnetic order. We believe that the large *k*_*z*_ broadening of the samples, which indeed manifests as “shadowing” of the electronic structure, combined to the strongly varying matrix elements (as shown in the Supporting Information) might be the reasons behind the apparent discrepancies. It is still worth mentioning that, although transport, ARPES, and DFT results indicate that TaCoTe_2_ realizes a possible AFM_*z*_ order, neutron scattering experiments would be also crucial to conclusively determine the precise magnetic ground state, but it is beyond the current scope of this work.

Together with the identification of the magnetic order in TaCoTe_2_, our data demonstrate that SOC plays an important role in opening energy gaps at the Dirac points. This can be seen in the AFM_*z*_ electronic structure calculations of Figure 2b, where D_1,2_ develops energy gaps right at the Fermi energy, while no gap is observed in D_3_. Such gaps are candidate *k*-space loci to enable topologically non-trivial behavior.^33−36^ We also experimentally detect such gaps: we show a zoom around D_1_ and D_3_ (Figure 3a), the intensity curvature plot in Figure 3b,^37^ and the extracted energy-distribution curved across the X′ point and exactly across D_1_ (panels c and d of Figure 3). The energy profile at the X′ point D_3_ does not show any gap but rather a single peak, consistent with the predicted antiferromagnetic behavior along the *z* direction (Figure 3c). As for D_2_, the small SOC-induced gap at the Fermi level predicted by DFT (≈20 meV) is more challenging to be observed with state-of-the-art experimental apparatuses. However, by fitting the dispersion with Lorentzian curves (purple markers in Figure 3a (see the Supporting Information for details), we estimate that the top of the band is located ∼25 ± 10 meV below the Fermi level. We stress that a proper quantification of this gap is difficult via ARPES, because it involves unoccupied states that are not accessible to ARPES. However, the value of the gap (∼25 ± 10 meV) adduced from the experimental data is a lower limit compatible with the calculated gap value. We also show in the Supporting Information how the observed peak is lower in binding energy compared to the Fermi level edge, extracted from energy distribution curves (EDCs) in a region without bands, i.e., at *k*_*x*_ = −0.5 Å^–1^.

**Figure 3:**
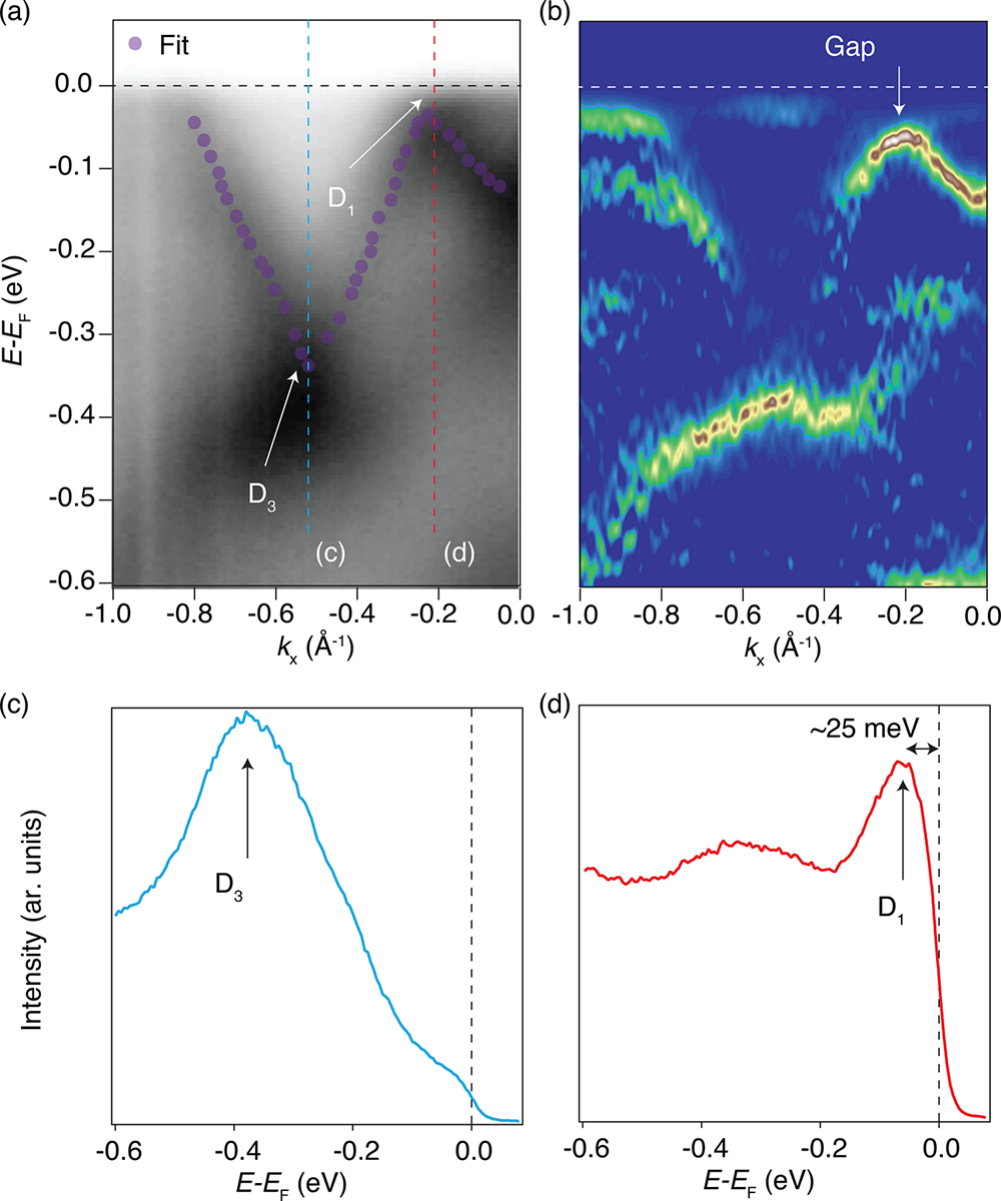
(a) Zoomed-in maps of the region near the Dirac dispersions D_1_ and D_3_. Purple dots are the positions of maximum intensity of the ARPES data, obtained by fitting the EDCs, and show the opening of a gap in D_1_ but not in D_3_. (b) Second derivative plot corresponding to the EDCs in panel a. (c) EDC across D_3_ at the X′ point, showing the absence of a gapped two-peak structure. (d) EDC across D_1_, showing the opening of a gap of at least 25 meV.

The presence of SOC gaps is of paramount importance for giving rise to exotic transport phenomena and, in particular, for the INHE in antiferromagnetic Dirac systems. The INHE arises from the field correction to the Berry curvature in the presence of an external electric field, and it has recently gained significant attention as a result of its intrinsic nature.^38−41^ Moreover, INHE has been attributed to band geometric quantities, like the quantum metric tensor, and it has been described as the quantum metric dipole-induced nonlinear Hall effect^40−44^ (see the Supporting Information for a mathematical derivation of the INHE).

We used DFT to compute the INHE (σ) for the monolayer TaCoTe_2_ in the AFM_*y*_ and AFM_*z*_ magnetic configurations. The NM state preserves both the  and  symmetry, and as a result, all of the components of the INHE vanish identically in this case. In the AFM phases, both  and  symmetries are broken, but the combined  symmetry results in non-vanishing INHE. Specifically, in the AFM_*z*_ phase, the relevant symmetry is , which forces the σ_*xyy*_ component to vanish, while the σ_*yxx*_ is non-zero. In contrast, the AFM_*y*_ structure hosts the  symmetry, which results in a vanishing σ_*yxx*_ and a non-zero σ_*xyy*_. The INHE is shown in Figure 4a for the AFM_*z*_ configuration and in Figure 4c for the AFM_*y*_ configuration. In both cases, the INHE vanishes identically inside the bandgap, develops peaks near the band edge, and decays rapidly when the chemical potential is shifted away. It exhibits an opposite sign for the electron and hole doping for both AFM_*z*_ and AFM_*y*_ order. The INHE value is found to be of the order of 1 mA/V^2^, which is comparable to the recently reported values in metallic antiferromagnets.^38−41^ Interestingly, the INHE is almost an order of magnitude larger for AFM_*z*_ compared to AFM_*y*_. Finally, given the direct link between the INHE and Λ_*n*_ (band resolved contribution to the INHE as defined in eq 4 of the Supporting Information), we present the distribution of the latter on the band structure in panels b and d of Figure 4. As expected, near the band edge, Λ_αβγ_^*n*^(*k*) has the maximum value and decays away from the band edge.

**Figure 4:**
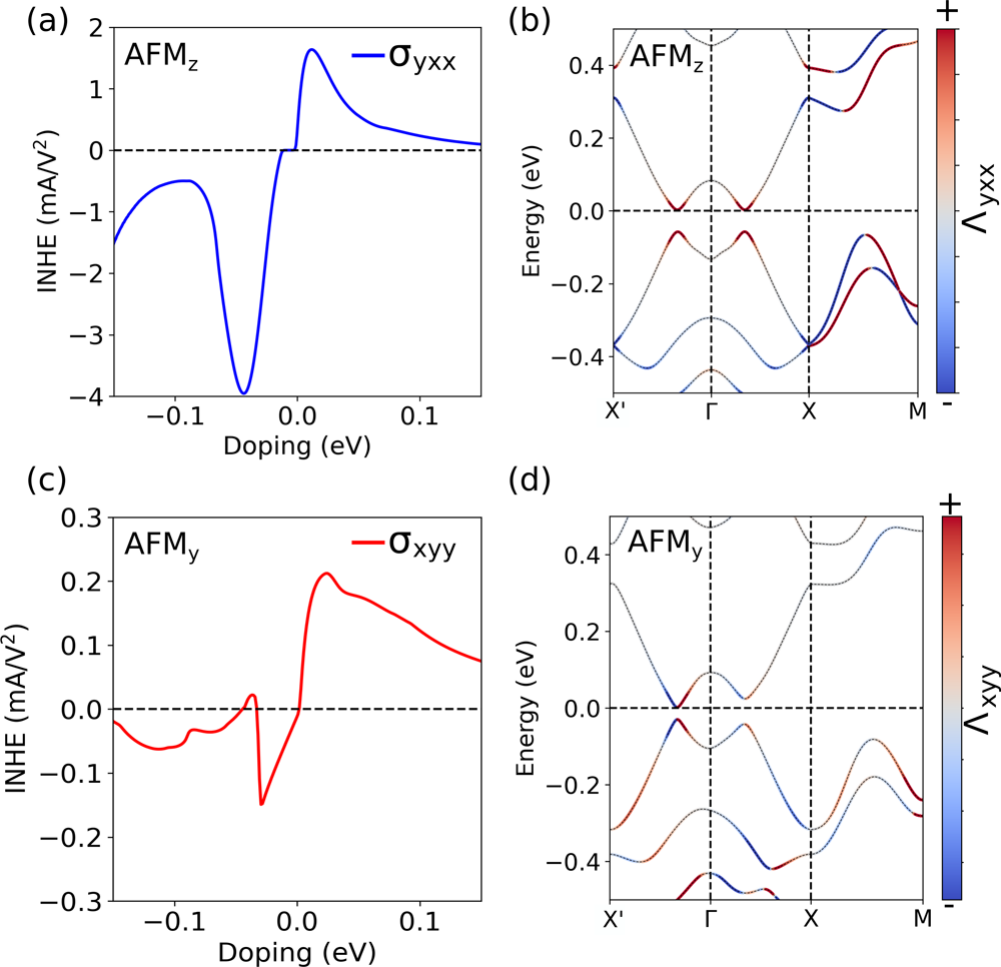
INHE and distribution of Λ_αβγ_^*n*^(*k*) on the band structure of TaCoTe_2_ in the (a and b) AFM_*z*_, and (c and d) AFM_*y*_ phases. Note that distinct symmetries of the two different magnetic configurations enforce different non-zero components of the INHE. Clearly, the INHE is highly sensitive to the Néel vector orientation.

In conclusion, we demonstrate that TaCoTe_2_ is a magnetic Dirac system, which hosts SOC-driven bandgaps at the Fermi level. The combination of SOC effects, magnetism, and time-reversal symmetry breaking is found to generate a non-vanishing INHE, which influences to the transport properties of the system. The INHE in TaCoTe_2_ is highly sensitive to the direction of the Néel vector of the AFM order, opening a novel pathway for using this compound in dissipationless electronics and spintronics. Our study indicates that TaCoTe_2_ would provide a promising new material platform for exploring the interplay of Dirac fermiology, SOC, magnetism, and topology.

## Supporting Information Available

Additional experimental data (both transport measurements and additional ARPES) and also equations useful to understand the theoretical results shown in the main text (PDF)
