# Supplementary material for: Discovery of a Magnetic Dirac System with a Large Intrinsic Nonlinear Hall Effect
Source: Nano Lett. 2023 Jan 23;23(3):902–7. doi: 10.1021/acs.nanolett.2c04194 (PMC10064332; doi:10.1021/acs.nanolett.2c04194)
Supplement: Supplementary file 1 — nl2c04194_si_001.pdf [file nl2c04194_si_001.pdf]

Discovery of a magnetic Dirac system with large intrinsic non-linear Hall effect

Federico Mazzola,[*,†] Barun Ghosh,[*,¶] Jun Fujii,[†] Gokul Acharya,[§] Debashis Mondal,[†] Giorgio Rossi,[‖] Arun Bansil,[¶] Daniel Farias,[⊥] Jin Hu,[§] Amit Agarwal,[@] Antonio Politano,[△] and Ivana Vobornik[†]

†CNR-IOM TASC Laboratory, Area Science Park, I-34149 Trieste, Italy

‡Department of Molecular Sciences and Nanosystems, Ca' Foscari University of Venice, 30172 Venice, Italy

¶Department of Physics, Northeastern University, Boston, Massachusetts 02115, USA

§Department of Physics, University of Arkansas, Fayetteville, AR 72701

‖University of Milano, I-20133 Milano, Italy

⊥Departamento de Física de la Materia Condensada, Universidad Autónoma de Madrid, 28049, Madrid, Spain

#Instituto "Nicolás Cabrera" and Condensed Matter Physics Center (IFIMAC), Universidad Autónoma de Madrid, 28049, Madrid, Spain

@Department of Physics, Indian Institute of Technology Kanpur, Kanpur 208016, India

△Department of Physical and Chemical Sciences, University of L' Aquila, via Vetoio 67100 L' Aquila (AQ), Italy

E-mail: federico.mazzola@unive.it; barunghosh02@gmail.com

January 21, 2023

$\mathbf{AFM}_z$ and $\mathbf{AFM}_y$ differences

The main differences between the AFM_z and AFM_y orders are expected due to the non-symmorphic symmetries, which are characterized by the combination of $\mathcal{P}$, a two-fold screw rotation symmetry along the y-axis $\tilde{\mathcal{C}}_{2y} = \{\mathcal{C}_{2y}|\frac{1}{2}\frac{1}{2}0\}$, and a glide mirror symmetry perpendicular to the y-axis $\tilde{\mathcal{M}}_y = \{\mathcal{M}_y|\frac{1}{2}\frac{1}{2}0\}$. In particular, the AFM_z order preserves the $\tilde{\mathcal{C}}_{2y}$, while the AFM_y order hosts $\tilde{\mathcal{M}}_y$. This difference is not only important for transport properties, but it is responsible for giving rise to significantly different k-resolved electronic dispersion.

Photon energy ARPES and light polarization dependence

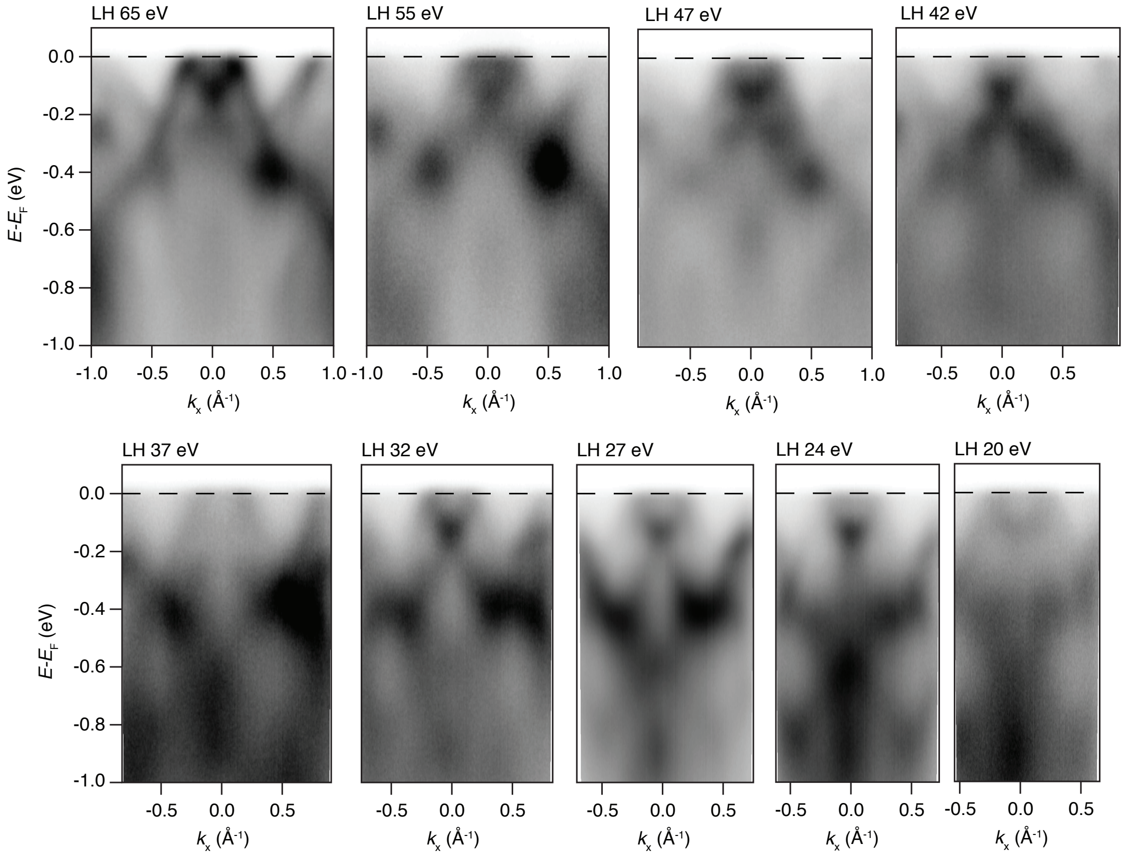

Figure 1: ARPES energy-momentum spectra collected wit linear horizontal light polarization and for different photon energies, as indicated in the panels. The main difference is a shift of the spectral weight in the spectra, but no significant change in the electronic structure can be observed. This confirms the lack of k_z dispersion, thus a two dimensional character for the electronic structure of $TaCoTe_2$.

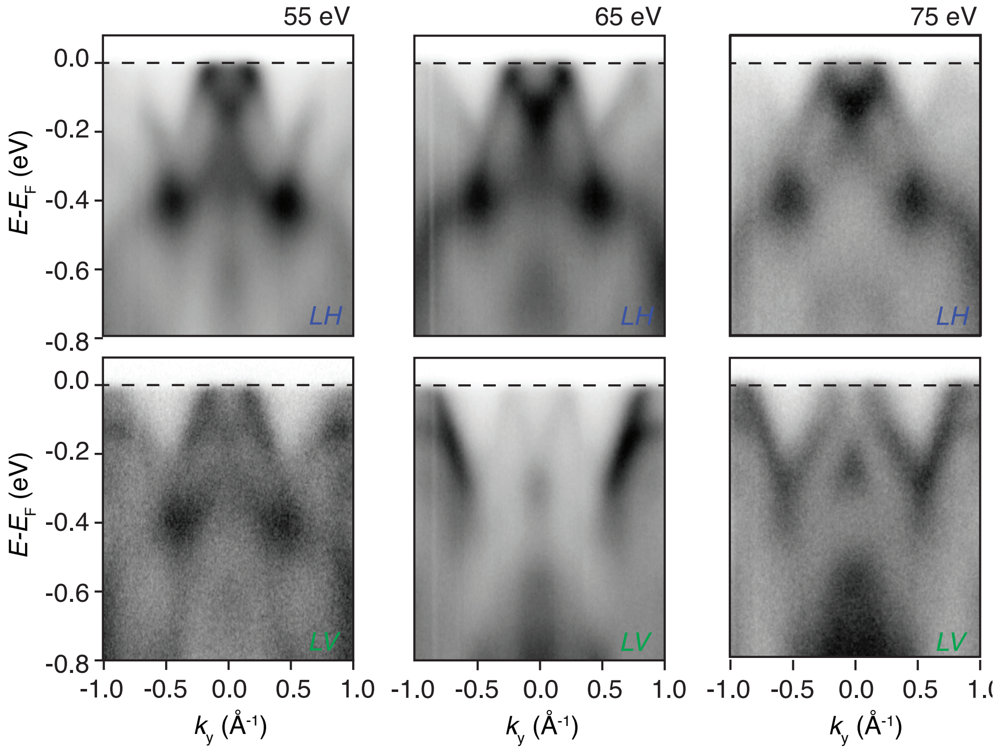

Figure 2: ARPES energy-momentum spectra at selected photon energies (where the intensity was prominent) and for both LH and LV. The main differences can be brought back to a redistribution of the spectral weight, indicating a mixed orbital character for the electronic structure collected within the energy region presented.

Sample growth and characterization

$TaCoTe_2$ single crystals were grown by a chemical vapor transport method . The stoichiometric mixture of Ta, Co, and Te powders was sealed in a quartz tube with $TeCl_4$ being used as transport agent. Thin plate-like single crystals with metallic luster can be obtained via the chemical vapor growth with a temperature gradient of 900°C - 750°C. The composition and structure of the crystals were checked by Energy-dispersive x-ray spectrometer and x-ray diffractometer respectively.

Methods and samples preparations

The samples were cleaved in ultrahigh vacuum at the base pressure of 1×10^{-10} mbar. The ARPES measurements were performed at the NFFA APE-Low Energy beamline, at 77 K, by using a Scienta DA30 hemispherical analyzer with energy and momentum resolutions better than 12 meV and 0.02 Å^{-1}, respectively.

ARPES Fitting Details

The electronic structure of Fig.3a of the main text has been fitted by using Lorentzian curves convoluted by a Gaussian, which accounts for the energy resolution of the instrument. For performing the fit, the ARPES spectra have been decomposed in energy distribution curves (EDCs) and each EDC has been fitted the way here described.

Details of the *ab-initio* calculations

The density functional theory based $ab - initio$ calculations were performed using the generalized gradient approximation framework (GGA-PBE) as implemented in VASP code .[1–4] A k-grid of 8×8×1 was used for the BZ integration. The kinetic energy cutoff for the plane wave basis was set to 400 eV. The Wannier function-based tight binding modeling is done by considering the s and d orbitals of Ta, the p orbital of Te, and the d orbitals of Co .[5,6]

Temperature Dependent Resistivity and Magnetization Data

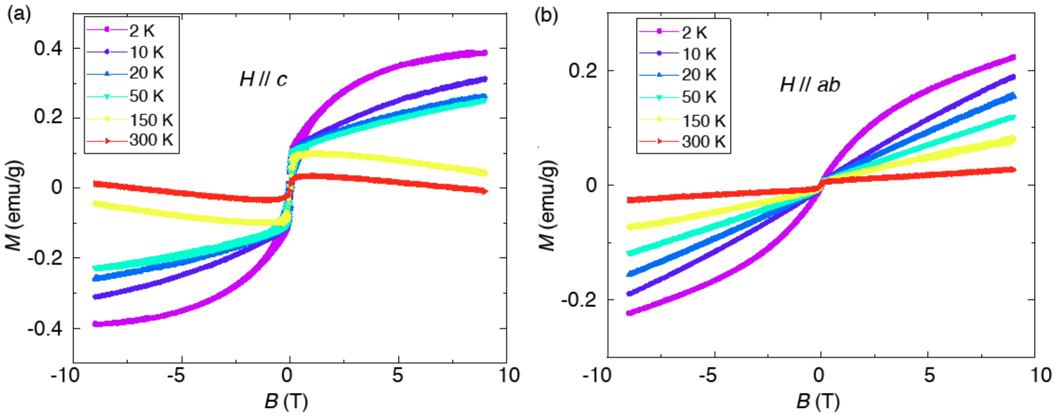

Figure 3: Temperature dependent magnetization data for $TaCoTe_2$ for the applied magnetic field along (a) $B||c$ direction, and (b) $B||ab$ direction. Clearly, the isothermal magnetization displays a typical moment polarization behavior for all measured temperatures up to 300 K, under both out-of-plane ($B||c$) and in-plane ($B||ab$) magnetic field orientations. Such behavior signatures the presence of magnetic order in the system. The out-of-plane magnetization is roughly twice as large as the in-plane one, suggesting that the easy axis is mainly along the out-of-plane direction.

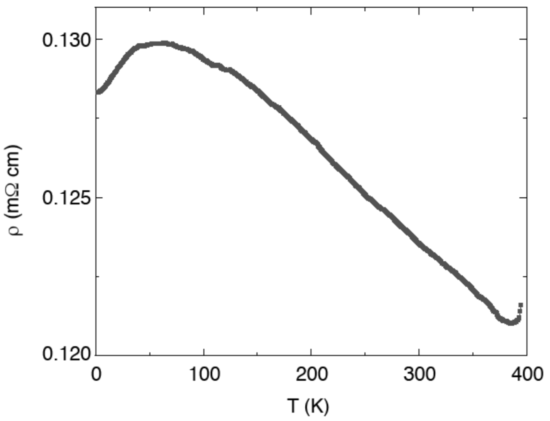

Figure 4: Temperature dependent resistivity of $TaCoTe_2$. The resistivity does not strongly vary with the temperature. Overall, it displays an non-metallic transport with resistivity increases upon cooling, followed by a resistivity down turn around 50 K. A transition-like behavior characterized by resistivity upturn is observed around 380K, which is reproducible in multiple samples with slight variation in upturn temperature.

INHE and Berry curvature connection

In a simple semi-classical model, INHE can be understood from the presence of a finite Berry connection polarizability (BCP) in the system. The BCP for the the n'th band can be expressed as,

$$G^n_{ab}(\mathbf{k}) = 2\mathrm{Re}\sum_{m\neq n} \frac{A^{nm}_a(\mathbf{k})A^{mn}_b(\mathbf{k})}{(\epsilon_n(\mathbf{k}) - \epsilon_m(\mathbf{k}))}, \tag{1}$$

where, $A^{nm}_a = \langle u_n | i\partial_a | u_m\rangle$ is the interband Berry connection, and $|u_n\rangle$ are the unperturbed Bloch states with band energies ϵ_n. In the presence of an external electric field (E_b), the net generated Berry connection can be obtained as,

$$A^E_a(\mathbf{k}) = G_{ab}(\mathbf{k})E_b \tag{2}$$

The field-induced Berry curvature $\Omega_E(k) = \nabla \times A^E(k)$ results in a non-linear Hall-like response. The most important feature of this response is that it is intrinsic, and unlike the Berry curvature dipole induced nonlinear Hall conductivity, INHE does not depend on the relaxation time (τ). The final expression for the INHE is given by,

$$\sigma_{\alpha\beta\gamma} = \int \frac{d^3k}{(2\pi)^3} \sum_n \Lambda^n_{\alpha\beta\gamma}(\mathbf{k}) \frac{df(\epsilon_n(\mathbf{k}))}{d\epsilon_n(\mathbf{k})} \ . \tag{3}$$

where, $f(\epsilon_n)$ is the Fermi Dirac distribution function. The momentum-resolved $\Lambda^n_{\alpha\beta\gamma}$ (we suppress the argument $\mathbf{k}$ for brevity) is expressed in terms of the interband Berry connection and the velocity operator (v_n) as,

$$\Lambda^n_{\alpha\beta\gamma} = 2e^3 \sum_m^{\epsilon_n\neq\epsilon_m} \Re[\frac{v^\alpha_n A^\beta_{nm} A^\gamma_{mn}}{(\epsilon_n - \epsilon_m)} - \frac{v^\beta_n A^\alpha_{nm} A^\gamma_{mn}}{(\epsilon_n - \epsilon_m)}] \ . \tag{4}$$

Fermi level and observed gap

The observed peak in the electronic structure, which forms the top of the bands which develop a gap, is lower in binding energy compared to the Fermi level edge, extracted from EDCs in a region without bands, i.e. at k_x=−0.5 Å^{-1}. This is visible in the figure below.

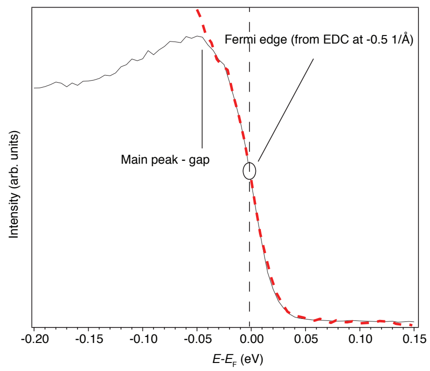

Figure 5: The red dashed line has been extracted from EDc around k_x=−0.5 Å^{-1}, where there are no bands in the ARPES data. This allows us to determine the leading edge of the Fermi level as a calibration. We observe that the peak of the electronic structure discussed in the main text, formed by the band which delevlops a gap, is shifted away from the edge. The latter also, despite the increase (as observed in the figure and caused by the presence of additional bands at lower binding energies) is still visible.

References

(1) Kohn, W.; Sham, L. J. Self-Consistent Equations Including Exchange and Correlation Effects. *Phys. Rev.* **1965**, *140*, A1133–A1138.

(2) Perdew, J. P.; Burke, K.; Ernzerhof, M. Generalized Gradient Approximation Made Simple. *Phys. Rev. Lett.* **1996**, *77*, 3865–3868.

(3) Kresse, G.; Furthmüller, J. Efficient iterative schemes for ab initio total-energy calculations using a plane-wave basis set. *Phys. Rev. B* **1996**, *54*, 11169–11186.

(4) Kresse, G.; Joubert, D. From ultrasoft pseudopotentials to the projector augmented-wave method. *Phys. Rev. B* **1999**, *59*, 1758–1775.

(5) Marzari, N.; Vanderbilt, D. Maximally localized generalized Wannier functions for composite energy bands. *Phys. Rev. B* **1997**, *56*, 12847–12865.

(6) Pizzi, G.; Vitale, V.; Arita, R.; Blügel, S.; Freimuth, F.; Géranton, G.; Gibertini, M.; Gresch, D.; Johnson, C.; Koretsune, T. et al. Wannier90 as a community code: new features and applications. *Journal of Physics: Condensed Matter* **2020**, *32*, 165902.
